# The CpG Dinucleotide Adjacent to a κB Site Affects NF-κB Function through Its Methylation

**DOI:** 10.3390/ijms18030528

**Published:** 2017-03-01

**Authors:** Tao Wang, Jinge Li, Ke Ding, Li Zhang, Qiuru Che, Xiuming Sun, Yumeng Dai, Wei Sun, Meiying Bao, Xiaochun Wang, Liquan Yang, Zhiwei Li

**Affiliations:** The Center for Combinatorial Chemistry and Drug Discovery, School of Pharmaceutical Science, Jilin University, Changchun 130012, China; wangtaodluf@163.com (T.W.); lijg2012772002@163.com (J.L.); m15250953673@163.com (K.D.); m13844142327@163.com (L.Z.); 15843056163@163.com (Q.C.); sunxiuming222@163.com (X.S.); daiyumeng_jlu@163.com (Y.D.); sunwei1067@163.com (W.S.); baomeiying1984@126.com (M.B.); sirzhe@outlook.com (X.W.); yanglq@jlu.edu.cn (L.Y.)

**Keywords:** NF-κB, consensus sequence, CpG islands, methylation

## Abstract

NF-κB is an important transcription factor that plays critical roles in cell survival, proliferation, inflammation, and cancers. Although the majority of experimentally identified functional NF-κB binding sites (κB sites) match the consensus sequence, there are plenty of non-functional NF-κB consensus sequences in the genome. We analyzed the surrounding sequences of the known κB sites that perfectly match the GGGRNNYYCC consensus sequence and identified the nucleotide at the -1 position of κB sites as a key contributor to the binding of the κB sites by NF-κB. We demonstrated that a cytosine at the -1 position of a κB site (-1C) could be methylated, which thereafter impaired NF-κB binding and/or function. In addition, all -1C κB sites are located in CpG islands and are conserved during evolution only when they are within CpG islands. Interestingly, when there are multiple NF-κB binding possibilities, methylation of -1C might increase NF-κB binding. Our finding suggests that a single nucleotide at the -1 position of a κB site could be a critical factor in NF-κB functioning and could be exploited as an additional manner to regulate the expression of NF-κB target genes.

## 1. Introduction

Transcription factor NF-κB regulates the expression of hundreds of genes involved in cell survival, proliferation, inflammation, cancer and other pathophysiological conditions. In mammalian cells, there are five NF-κB family members, including RelA/p65, RelB, c-Rel, P50, and p52 [1]. These molecules function as active homodimers or heterodimers to regulate gene expression after binding to specific DNA sequences [2]. Among these NF-κB molecules, the most studied is p65/p50 heterodimer, the classical NF-κB [1].

The classical NF-κB consensus sequence, i.e., the DNA sequences that bind to p65/p50 heterodimers, was originally identified as GGGRNNYYCC (where N is any base, R is a purine, and Y is a pyrimidine) by statistical analysis of the known NF-κB binding sites (κB sites) [3]. The studies on the crystal structures of p65 and/or p50 provided further evidence supporting the consensus sequence, and identified 5′-GGGRN-3′ and 5′-YYCC-3′ as the binding sites for p50 and p65, respectively [4,5,6,7]. Research with other technologies such as System Evolution of Ligands by Exponential Enrichment (SELEX) and Protein-Binding Microarray (PBM) has confirmed the above finding and extended our knowledge in the consensus sequences [8,9]. Using PBM, the DNA-binding preferences of ten NF-κB dimers to a wide-ranging set of 3285 potential κB site sequences were examined systematically, and the DNA-binding preferences were quantitated and transformed into z-scores [9].

It was reported that different nucleotides within the κB site could differentially affect NF-κB function. For example, the nucleotides at the +6 position could contribute to the NF-κB binding specificity of other molecules. When it was an adenine, the bound p65/p65 homodimer recruited interferon regulatory factor 3 (IRF3) in order to activate the target gene expression [10]. When it was a cytosine or a thymine, the bound p65/p65 homodimer recruited yet to be identified factor(s) in order to activate the target gene expression [10]. In comparison, a p52/p52 homodimer could present different kinetic stability and conformation when complexed with B-cell CLL/lymphoma 3 (Bcl3) and thereafter could recruit different co-activators or co-inhibitors when binding to consensus sequences with a different nucleotide at +6 position [11]. When it was a cytosine or guanine, the p52/p52/Bcl3 trimer activated target gene expression [11]. When it was an adenine or thymine, interestingly, the trimer inhibited target gene expression [11].

In mammalian cells, the C5 methylation at CpG sites is a significant contributor in the epigenetic regulation of gene transcription. In the genome, CpG dinucleotides in clusters are called CpG islands (CGIs) [12,13]. CGIs predominantly overlap gene promoter regions and are typically non-methylated [14]. Methylation of CGIs robustly represses the gene transcription [15]. It is still unclear whether DNA methylation in CpG-poor regulatory regions will affect gene transcription, although it is believed that DNA methylation around transcription binding sites or within CGIs in the promoter region will prevent the binding of transcription factors [15,16,17].

Although the majority of p65/p50 binding sequences match the consensus sequence GGGRNNYYCC, the consensus sequences in the genome may not always be functional upon NF-κB activation [7]. This functional difference could occur even between two adjacent κB sites [18], or two identical κB sites surrounded by different DNA sequences [19]. These findings suggest that the DNA sequences surrounding the κB sites could affect NF-κB binding or function in certain situations. Although it was reported that DNA methylation at the CpG sites, within or surrounding transcription factors-binding sequences, would inhibit the binding between DNA and transcription factors, it is still unclear whether methylation of a single nucleotide flanking the transcription factor-binding sequence would lead to the same consequence [20]. We report here that the methylation of the cytosine immediately adjacent to the κB sites at the 5’-end, designated as -1C, could inhibit the binding of the κB sites with NF-κB, and thereafter the function of NF-κB, and therefore, provide an extra regulatory manner in the expression of NF-κB target genes.

## 2. Results

### 2.1. Low Frequency of a Cytosine at the -1 Position of the κB Sites

**Figure 1:**
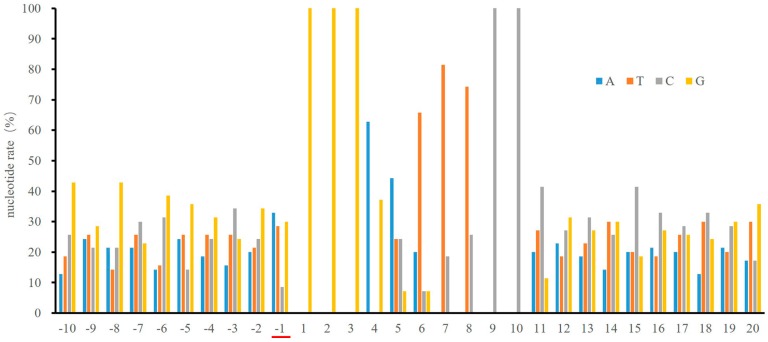
A cytosine appears less frequently at the -1 position than any of other three bases. The surrounding sequences of a total of 70 functional human κB sites were analyzed. The bases of the NF-κB consensus sequence were numbered as 1–10, the upstream sequences were numbered from -1 and up. The analysis result of the sequences from −10 to +20 is shown here.

The finding that NF-κB selectively functions at some but not all of its consensus sequences in the genome suggests that other factor(s), in addition to the consensus sequence itself, might affect either NF-κB binding or its activity after binding to DNA [18,19]. In order to identify these potential factors, we compared DNA sequences 50 bps upstream and downstream the κB sites (Table S1) that were experimentally identified as GGGRNNYYCC. As shown in Figure 1, the frequency of a -1C κB site is substantially lower than that of any of other three nucleotides.

### 2.2. NF-κB DNA Binding Capacity is not Affected by the Nucleotide at the -1 Position

**Figure 2:**
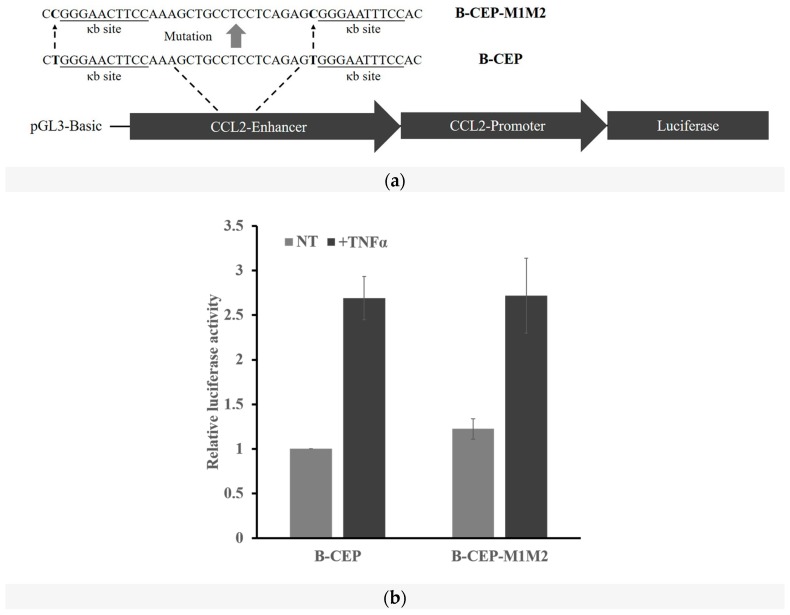
The -1C does not affect NF-κB function. (**a**) Schematic maps of reporter constructs B-CEP and B-CEP-M1M2. The κB site sequences were underlined. The T/C mutations were indicated with dotted arrows; (**b**) Luciferase activity of HEK 293T cells transfected with B-CEP or B-CEP-M1M2, respectively, with or without TNFα (10 ng/mL) stimulation for 18 h. NT, not treated. Data shown are means ± S.D. of results from three independent experiments.

We wondered whether the reduced -1C frequency, as shown in Figure 1, might implicate any biological consequence. We therefore used C-C motif chemokine ligand 2 (*CCL2*) gene enhancer/promoter as a model to investigate the role of -1C in NF-κB function (Figure 2a). *CCL2* is a well-known NF-κB target gene and there are two functional κB sites in its enhancer. NF-κB activation was monitored by luciferase assay of HEK 293T cells transfected with pGL3-Basic-CCL2-enhancer-promoter (B-CEP) or pGL3-Basic-CCL2-enhancer-promoter-mutation1-mutation2 (B-CEP-M1M2), respectively, followed by tumor necrosis factor alpha (TNFα) treatment for 18 h. As shown in Figure 2b, there was no significant difference in luciferase activity between B-CEP transfected and B-CEP-M1M2 transfected cells, suggesting that the -1T to -1C mutation had no impact on NF-κB function.

### 2.3. DNA Methylation at the -1C Inhibits DNA Binding and Thereafter the Function of NF-κB

**Figure 3:**
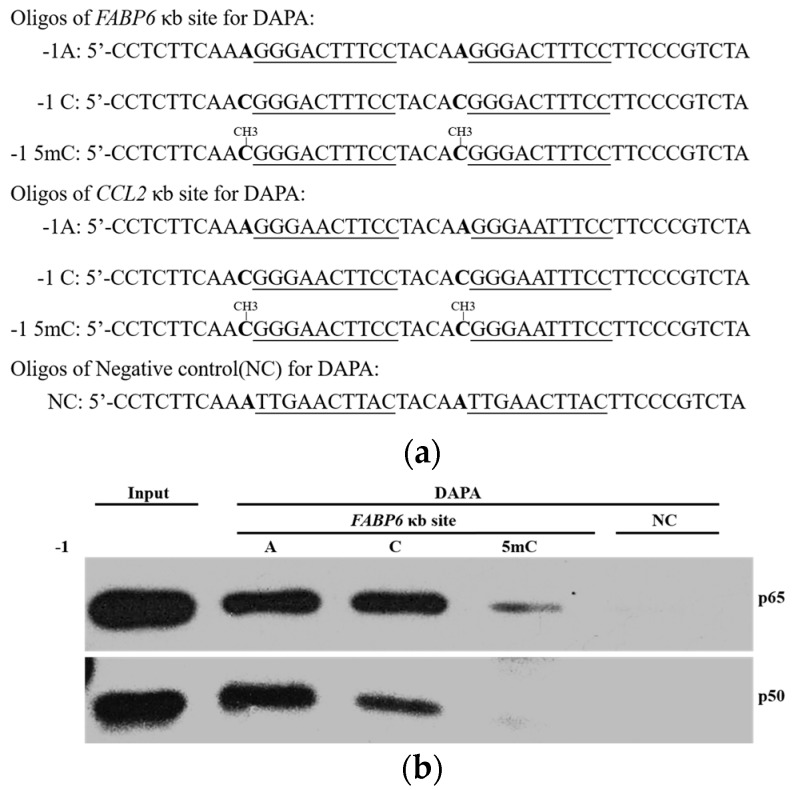

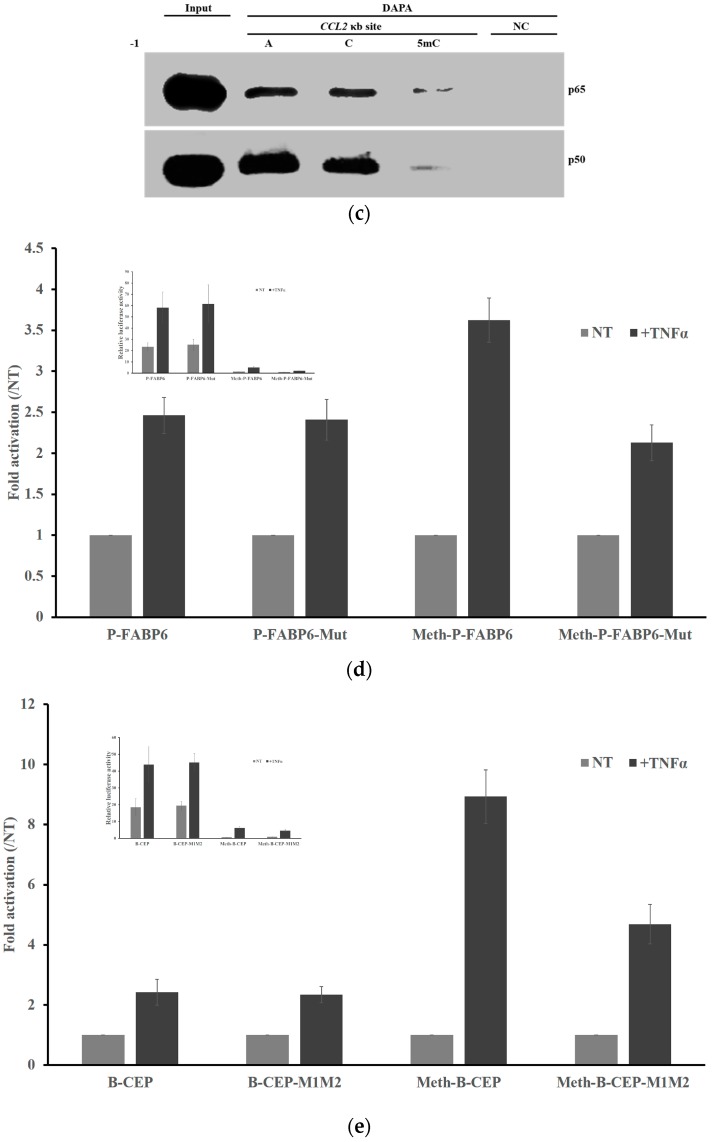
Methylation of the -1C nucleoside inhibited the binding and function of NF-κB. (**a**) Sequences of the oligonucleotides with tandem *FABP6* or *CCL2* κB sites used in DAPA. The κB site sequences were underlined, the nucleotides at -1 position were in bold; (**b**,**c**) DAPA using *FABP6* (**b**) or *CCL2* (**c**) oligonucleotides shown above and lysates of HEK 293T cells treated with TNFα (10 ng/mL) for 30 min. Oligonucleotides-bound NF-κB was detected by Western blot analysis using antibodies against p65 or p50, respectively. NC, negative control oligonucleotide. Input, 2% of the whole cell lysate used in DAPA; (**d**,**e**) Reporter assays of TNFα (10 ng/mL, 18 h) treated HEK 293T cells transfected with plasmids shown in the figures. Fold activations are the luciferase activities of TNFα treated group normalized to their untreated controls. The inserts shown are the relative luciferase activities of all eight reporters in each experiment. (**b**,**c**) are the results of one of three experiments. Data shown in (**d**,**e**) are means ± S.D. of the results from at least three independent experiments.

Since -1C does not affect NF-κB function, we then asked whether -1C methylation would substantially reduce the binding affinity between DNA and NF-κB. We carried out DNA affinity precipitation assays (DAPAs) using two tandem κB sites of fatty acid binding protein 6 (*FABP6*) gene (Figure 3a). As shown in Figure 3b, the amount of p65 and p50 binding to the oligonucleotides was dramatically reduced when the oligonucleotides had a 5-methylcytosine (5mC), rather than an adenosine or a cytosine, at the -1 position. The NF-κB binding capacity was not significantly affected between -1A and -1C oligonucleotides. This finding suggested that it was methylation, not nucleotide replacement itself that caused the decreased NF-κB binding capacity. To verify this finding with other κB sites, we replaced the κB site of *FABP6* in the oligonucleotides used in DAPAs with that of *CCL2* (Figure 3a). DAPAs results demonstrated that it was true that only 5mC, not mutation of the nucleotide itself, could inhibit the binding between NF-κB and the oligonucleotides (Figure 3c).

To determine whether reduced NF-κB binding would affect its activity in cells, we cloned the *FABP6*-1A and -1C oligonucleotides used in DAPAs into the luciferase report plasmid pGL3-Promoter, which resulted in P-FABP6 and P-FABP6-M (with A to C mutation at -1C position), respectively. In vitro methylation of *FABP6* κB site containing plasmids (P-FABP6, P-FABP6-M) and *CCL2* κB site containing plasmids (B-CEP, B-CEP-M1M2) with CpG methyltransferase M.SssI allowed us to determine whether the single 5mC modification would affect the function of NF-κB in living cells. We transfected HEK 293T cells with the above four plasmids and their methylated counterparts, and measured their luciferase activity with or without TNFα stimulation. As shown in Figure 3d,e, the ratios between luciferase activities in cells treated with or without TNFα were not affected by the A to C mutation (P-FABP6 vs. P-FABP6-Mut in Figure 3d and B-CEP vs. B-CEP-M1M2 in Figure 3e), but were significantly suppressed by the single A to 5mC mutation (Meth-P-FABP6 vs. Meth-P-FABP6-Mut in Figure 3d and Meth-B-CEP vs. Meth-B-CEP-M1M2 in Figure 3e). These assays suggested that 5mC, not the mutation of the nucleotide itself, could inhibit the κB site function. Interestingly, TNFα activation of NF-κB led to a more dramatic increase in the luciferase activity in cells transfected with methylated plasmids than that with non-methylated plasmids (Meth-plasmids vs. plasmids), although the absolute luciferase activity was dramatically lower than that with non-methylated plasmids.

### 2.4. A -1C κB Site Is Preferably Located in a CGI to Avoid Methylation

**Figure 4:**
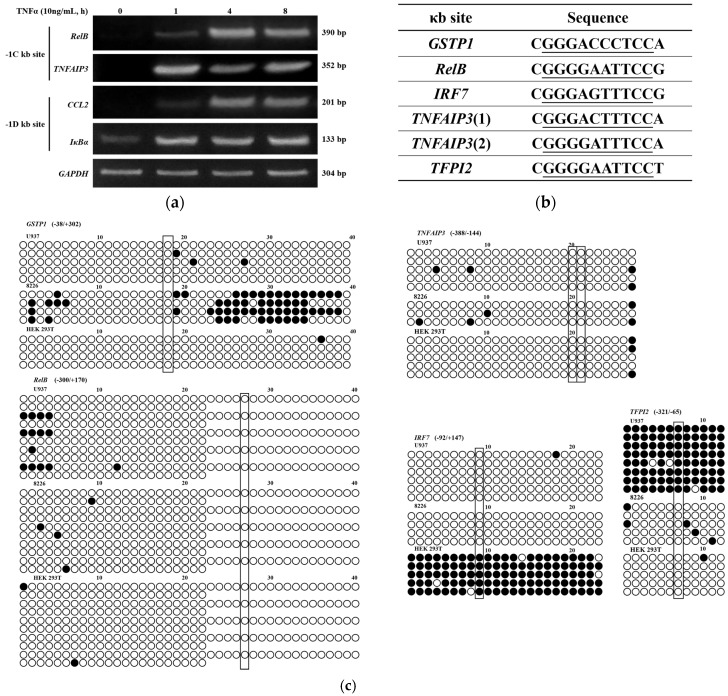

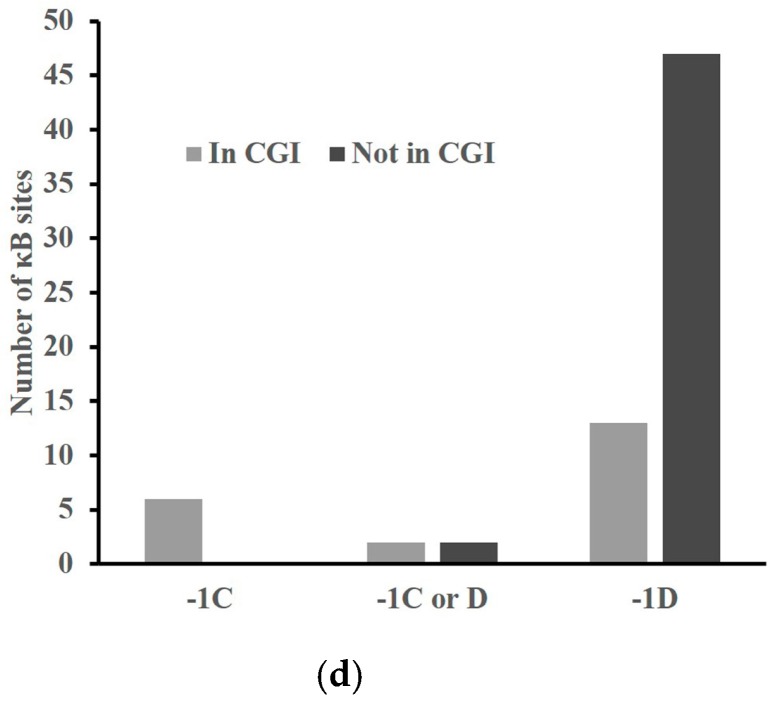
Hypo-methylation of the κB site and its surrounding sequences is essential for the function of NF-κB. (**a**) Expression of -1C κB site containing genes (-1C genes) (*RelB* and *TNFAIP3*) or -1D (D = A, T, G) genes (*CCL2* and *IκBα*) in U937 cells upon TNFα (10 ng/mL) stimulation. *GAPDH* expression was used as an internal control. Data shown are the results of one of three experiments; (**b**) -1C κB sites used in this study. *GSTP1*, glutathione S-transferase pi 1. The κB site sequences were underlined. (**c**) Methylation status of κB sites and their surrounding sequences of -1C genes (Figure 3a) analyzed by bisulfite sequencing PCR (BSP) in U937, 8226 and HEK 293T cells. The CpGs at -1 position of κB sites are outlined. Methylated and non-methylated CpG sites were shown as solid or empty circles, respectively; (**d**) All -1C κB sites are located in CGIs. The κB sites in CGIs and their surrounding sequences (−500 to +500) of all 70 κB sites shown in Figure 1 were analyzed using the MethPrimer program with the following parameters: Island size >100, GC Percent > 50.0, Obs/Exp > 0.6.

As shown above, -1C methylation inhibited NF-κB binding and the κB site function, suggesting that the gene transcription regulated by -1C containing κB site will be dramatically affected by the methylation of -1C within the sequences. However, all these -1C genes were reported to be functional in response to NF-κB activation [21,22,23,24,25]. To understand this controversy, we examined the expression of several genes with or without a cytosine immediately adjacent to their κB sites. As shown in Figure 4a, upon TNFα activation of NF-κB in U937 cells, transcription of -1C genes (*RelB* and TNFα-induced protein 3 (*TNFAIP3*)), or -1D genes (*CCL2* and Inhibitor of NF-κB alpha (*IκBα*)) all increased, suggesting that the promoters/enhancers with -1C κB sites were hypo-methylated in order to maintain their functions.

To directly detect the methylation status of -1C genes, we studied the CpG dinucleotide methylation of all six -1C κB sites (Figure 4b), identified from the 70 κB sites analyzed, in U937, 8226 and HEK 293T three cell lines by bisulfite sequencing PCR (BSP). As shown in Figure 4c, among all the six κB sites examined, only tissue factor pathway inhibitor 2 (*TFPI2*) κB site in U937 cells and interferon regulatory factor 7 (*IRF7*) κB site in HEK 293T cells were hyper-methylated. This confirmed that -1C genes are usually hypo-methylated.

**Figure 6:**
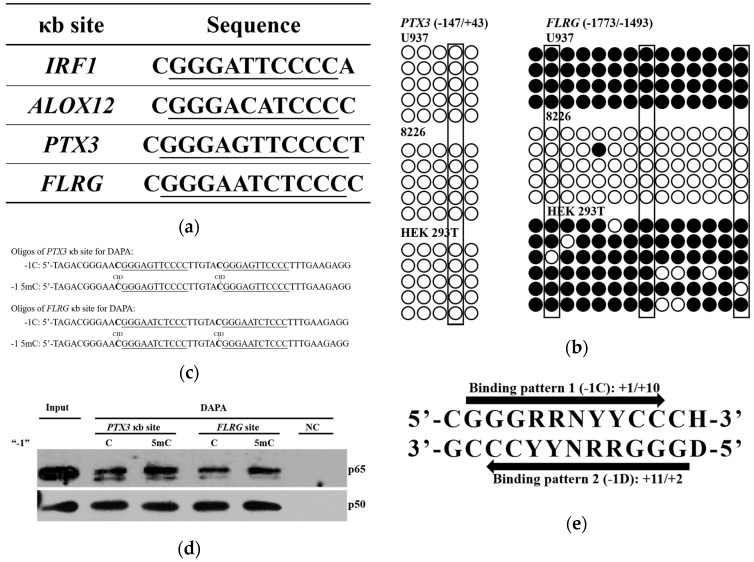
The -1C methylation might not affect NF-κB binding when there are other binding possibilities. (**a**) Sequences of -1C κB sites with multiple binding patterns. The κB site sequences were underlined; (**b**) The methylation status of *PTX3* and *FLRG* κB sites and their surrounding sequences, analyzed by BSP in U937, 8226 and HEK 293T cells. The CpGs at -1 position of κB sites are outlined. Methylated and non-methylated CpG sites are shown as solid or empty circles, respectively; (**c**) Sequences of the oligonucleotides with tandem *PTX3* or *FLRG* κB sites used in DAPA. The κB site sequences were underlined, the nucleotides at -1 position were in bold; (**d**) Increased binding capacities of p65 and p50 with oligonucleotides bearing 5mC residues at -1C position of κB sites. DAPA were carried out using oligonucleotides containing κB sites of *PTX3* or *FLRG* genes shown in Figure 6c and lysates of HEK 293T cells treated with TNFα (10 ng/mL) for 30 minutes. Oligonucleotide-bound NF-κB was detected by Western blot analysis using antibodies against p65 or p50, respectively. Data shown are the results of one of three experiments. NC, negative control oligonucleotide. Input, 2% of the whole cell lysate used in DAPA; (**e**) Possible κB sites in *PTX3* and *FLRG*. D = A, T, G; H = T, A, C.

It was well known that the CpG dinucleotides in CGIs are usually hypo-methylated. Given the few cases that the -1C κB sites tested above were hyper-methylated, we hypothesized that -1C κB sites would be located in CGIs. Hence, we used the MethPrimer program to predict the potential CGIs within the sequences 500 bps upstream and downstream of the 70 κB sites [26]. As expected, all -1C κB sites were located in CGIs whereas only 22% of the -1D κB sites (D = A, G, or T) in CGIs (Figure 4d). As shown in Figure 6a, among the 70 κB sites studied, there are 4 genes that have “-1C or D” κB sites. These κB sites could have (an) additional C(s) at the 3′ end, resulting in multiple NF-κB binding patterns. Depending on which set of nucleotides would be bound by NF-κB, a -1C κB site or a -1D κB site could appear. This type of κB sites seems to be located in CGIs or in non-CGIs equally. These findings suggested that -1C κB sites are evolutionarily conserved only when within CGIs.

### 2.5. Methylation Dramatically Affects the Function of -1C Genes In Vivo

**Figure 5:**
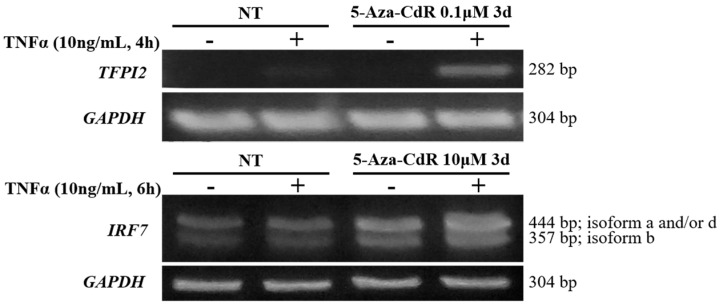
Demethylation restored the function of -1C κB sites. U937 and HEK 293T cells that do not express TFPI2 or IRF7, respectively, were treated with or without 5-Aza-CdR for three days. *TFPI2* or *IRF7* expression upon TNFα stimulation was then analyzed by reverse transcription PCR (RT-PCR). *GAPDH* expression was used as internal controls. Data shown are the results of one of three experiments.

Although methylation in CGIs does not occur in most cases, hyper-methylation in CGIs can happen in certain situations [27]. Therefore, it is critical to determine whether methylation in cells, not just in vitro, would be a key regulator in gene expression. As shown in Figure 5, upon TNFα activation of NF-κB, there were no significant changes in *TFPI2* and *IRF7* transcripts in U937 and HEK 293T cells, respectively. De-methylation with DNA methyltransferase inhibitor 5-Aza-2’-deoxycytidine (5-Aza-CdR) of whole genomic DNA [28,29,30], including CGIs in *TFPI2* and *IRF7* promoter regions, restored their response to NF-κB activation substantially. These results suggested that CpG methylation is a critical factor in regulating gene expression. Therefore, -1C provided an extra target in mediating gene expression.

### 2.6. Methylation of -1C Would Not Affect Target Gene Expression When There is Another NF-κB Binding Possibility

NF-κB specifically binds to the 10 nucleotide κB sites. When the κB sites are flanked immediately by a guanine nucleotide at the 5’ end or a cytosine nucleotide at the 3’ end, or when their complementary sequences can bind to NF-κB, the possibility for NF-κB binding is increased. Among the 70 κB sites analyzed, 23 κB sites have multiple possible sequences for NF-κB binding. Four out of these 23 κB sites could, but do not have to have a cytosine at the -1 position, depending on how the NF-κB binds to the sequences (Figure 6a). CGI statistical analysis indicated that among these four κB sites, interferon regulatory factor 1 (*IRF1*) and arachidonate 12-lipoxygenase, 12S type (*ALOX12*) have their κB sites in CGIs whereas pentraxin 3 (*PTX3*) and follistatin-related gene (*FLRG*) do not (Figure 4d).

Since CpG out of CGIs are likely to be methylated, we further analyzed by BSP, the methylation status of *PTX3* and *FLRG* regulatory sequences around their κB sites. As shown in Figure 6b, the *PTX3* κB sites and surrounding sequences were free of methylation in all three tested cell lines. In contrast, *FLRG* κB sites and surrounding sequences were hyper-methylated in U937 and HEK 293T cells. To determine whether the -1C methylation at the *FLRG* κB site would affect NF-κB binding, we carried out DAPA using oligonucleotides with *PTX3* or *FLRG* κB site (Figure 6c). Interestingly, -1C methylation at the *PTX3* or *FLRG* κB site did not inhibit but slightly increased NF-κB binding (Figure 6d). Both *PTX3* and *FLRG* upstream sequences contain at least two types of binding patterns as shown in Figure 6e; therefore, NF-κB could bind to κB sites with either pattern, which might be one reason for the tolerance of -1C methylation. Consistently, PBM assays demonstrated that the binding affinity of p65/p50 to the -1D κB sites (pattern 2 in Figure 6e) is higher than that to the -1C κB sites (pattern 1 in Figure 6e) in *PTX3* and *FLRG* genes [9]. Another reason for the slightly increased NF-κB binding might be that p65/p65 instead of p65/p50 could bind to the methylated κB sites since p65 binds to YYCC [4,5,6,9]. Indeed, it was p65, not p50, which showed increased the binding with the -1C methylated κB sites.

### 2.7. The -1C κB Sites Are Evolutionarily Conserved Only When Located in CGIs

The data shown above suggested that in most cases, -1C κB sites were functional only when located in CGIs. We therefore analyzed whether potential κB sites with -1C would be evolutionarily conserved. Due to the limited availability of the same genes in multiple vertebrates, the vascular cell adhesion molecule 1 (*VCAM1*), *CCL2* and *RelB* gene promoter and enhancer regions in several vertebrates were analyzed. In the human genome, VCAM1 and *CCL2* κB sites with a -1D are not located in a CGI; whereas *RelB* κB site with a -1C is located in a CGI. As shown in Table 1, for *RelB*, most vertebrates have their most proximate κB site in a CGI; whereas CGIs would not be needed by genes without a -1C, such as *VCAM1* and *CCL2*. Analysis of the data in Table 1 indicated that, as shown in Figure S2, the ratio between the -1C κB sites to all κB sites outside CGIs is substantially lower than that inside CGIs. These analyses suggested that a -1C κB site was evolutionarily conserved only when it was in a CGI.

## 3. Discussion

Transcription factor NF-κB regulates gene expression upon binding to the so called κB sites within the promoters/enhancers of target genes. Although its transcriptional specificity is controlled by the κB sites, NF-κB binding affinity to the κB sites and thereafter, functionality might also be affected by other factors since not all κB sites in the genome are functional. It was reported that a site-specific CpG methylation 12 bp downstream Sp1 binding site impaired the binding capacity of transcription factor Sp1 in the tumor suppressor *C/EBPδ* [31]. To understand whether other DNA sequences instead of κB sites would affect NF-κB binding and function, we analyzed the surrounding sequences of 70 κB sites that were experimentally verified to be functional. We found that a cytosine appears less frequently at the -1 position of κB sites than the other three bases do (Figure 1). The role of the κB site surrounding sequences in NF-κB binding and function has not been reported but has been implicated by others [3]. It was reported that methylation at the +10C of the human immunodeficiency virus-1 (*HIV-1*) κB site would inhibit its binding to NF-κB [20], suggesting that a guanine nucleotide is not preferred at the +11 position. Indeed, our analysis indicated that the frequency of a guanine existing at the +11 position of the κB sites is also lower than that of other three bases (Figure 1). These findings suggested that methylation of -1C or +11G might play a direct role in NF-κB binding and function. We therefore studied the role of -1C and its methylation in NF-κB binding and reporter gene expression regulated by NF-κB activation. We demonstrated that it was not the -1C itself, but its methylation, that affected the binding of NF-κB to κB sites, and thereafter, the NF-κB function (Figure 2 and Figure 3). Interestingly, although luciferase activities associated with methylated plasmids were dramatically lower than that with non-methylated plasmids, the relative increases of luciferase activities upon NF-κB activation were even higher when associated with the methylated plasmids than with non-methylated plasmids. The causes for the increase are unknown but it was not due to the methylation status change upon NF-κB binding since the methylation status was not changed in the surrounding sequences of *CCL2* κB sites upon TNFα or LPS stimulation (Figure S1). Hyper-methylation in the *CCL2* κB site surrounding sequences in U937 cells did not affect *CCL2* gene activation upon TNFα stimulation (Figure 4a and Figure S1), which might be because *CCL2* κB sites are in a CpG-poor regulatory region and because none of these CpG sites are adjacent to the κB sites.

Since all 70 κB sites were experimentally verified to be functional, the six κB sites, which have a -1C, should not be methylated at their -1C since methylation will dramatically inhibit NF-κB binding and function. To verify the functionality of the -1C κB sites, we analyzed the expression of genes with either a -1C or a -1D κB site. All the genes tested were up-regulated upon NF-κB activation by TNFα stimulation (Figure 4a), suggesting that the -1C κB sites were not methylated. Further analysis of all six κB sites in three cell lines indicated that among the total 18 κB sites analyzed, only *IRF7* κB site in HEK293 cells and *TFPI2* κB site in U937 cells were hyper-methylated (Figure 4c). This confirmed that -1C κB sites are usually hypo-methylated. Konduri et al. have reported that in many tumors, the promoter region of the tumor suppressor gene *TFPI2* is hyper-methylated [32,33,34,35,36,37,38]. Therefore, it is not a surprise that *TFPI2* promoter region was hyper-methylated in human malignant cell line U937 in our research. Similarly, it is understandable that the promoter region of *IRF7* is hyper-methylated in HEK 293T cells since IRF7 is an interferon regulator, whereas HEK 293T is a human embryonic kidney cell line rarely expressing interferons. The above findings suggested that methylation is the key factor in controlling NF-κB functionality in the expression of *TFPI2* and *IRF7* in U937 and HEK293 cells, respectively. Consistently, demethylation by 5-Aza-CdR did restore *TFPI2* and *IRF7* expression upon NF-κB activation by TNFα in U937 and HEK293 cells, respectively (Figure 5). Hypo-methylation of -1C κB sites suggested that all -1C κB sites should be in CGIs. This was confirmed by analyzing the sequences 500 bps upstream and downstream of the 70 κB sites using the MethPrimer program that predicts potential CGIs. In comparison, only 22% of the -1D κB sites are in CGIs (Figure 4d).

Since methylation would not usually occur in CGIs, the above finding led us to hypothesize that the -1C κB sites are evolutionarily conserved only when located in CGIs. To verify this hypothesis, we analyzed the promoter and enhancer regions of *VCAM1*, *CCL2* and *RelB* genes in multiple vertebrates. We found that for *RelB*, as a gene with a -1C κB site, its most proximate κB site is within a CGI in most vertebrates; whereas for *VCAM1* and *CCL2*, as genes with -1D κB sites, their most proximate κB sites are not in CGIs (Table 1). These findings not only verified our hypothesis, but are also consistent with our finding that -1C methylation may affect the binding of NF-κB to κB sites and therefore should be avoided.

In higher vertebrates, the predicted CGIs match the experimental non-methylated islands (NMIs) very well [39]. However, in lower vertebrates, there are big differences between the predicted CGIs and the experimental NMIs [39]. Therefore, in lower vertebrates, CGI maps may fail to accurately identify regions of non-methylated DNA [39]. This may explain why in lower vertebrates, some -1C κB sites are not in the CGIs. However, further investigation is necessary to determine whether they are in experimental NMIs.

CpG dinucleotide depletion over evolution was caused by inefficient base-excision repair and conversion from 5mc to thymine, which is mediated by activation-induced cytidine deaminase/apolipoprotein B mRNA-editing enzyme complex (AID/APOBEC) in vertebrates [16,40,41]. CGIs predominantly remain un-methylated and thereafter retain the expected CpG content [15,16]. This is consistent to the finding that all -1C κB sites are in CGI in human beings. Since the appearing frequency of thymine at the -1 position is similar to that of guanine and adenine (Figure 1), CpG depletion could be one reason, but not the only reason for the low frequency of a cytosine at the -1 position.

Depending on how NF-κB binds to κB sites, it is quite frequent that two or more κB sites could overlap one another. For example, *FLRG* gene has a 5′-CGGGAATCTCCCC-3′ upstream sequence, which contains two NF-κB consensus sequences: (1) 5′-GGGAATCTCC-3′; and (2) 5′-GGGAGATTCC-3′. Depending on which κB sites were used, a gene could have both -1C and -1D κB sites. In the case of *FLRG*, (1) is a -1C κB site; whereas (2) is a -1D κB site. As the NF-κB binding affinity is higher to -1D κB sites than to -1C κB sites in *PTX3* and *FLRG* [9], the methylation that inhibits binding to -1C κB sites might release more NF-κB to bind to -1D sites. Therefore, it is reasonable that -1C methylation would enhance NF-κB binding to -1D κB sites as shown in the cases of *PTX3* and *FLRG* (Figure 6d).

In this study, we demonstrated that -1C methylation could affect the binding affinity between NF-κB and κB sites, and thereafter, the pathophysiological function of -1C κB sites. To maintain the -1C κB site function, a -1C κB site is evolutionally conserved only when located in a CGI. Depending on the κB sites utilized, -1C methylation may reduce or enhance NF-κB binding affinity. Our study suggests that the epigenetic modification of a single nucleotide adjacent to the NF-κB consensus sequences would provide an extra regulatory manner to the expression of NF-κB target genes.

## 4. Materials and Methods

### 4.1. Cell Culture and Reagents

HEK 293T cells and U937 cells were cultured in DMEM and RPMI 1640 medium supplemented with 10% fetal bovine serum, respectively. Streptavidin-Agarose (S1638) and 5-Aza-CdR (A3656) were purchased from Sigma. Recombinant human TNFα (300-01A) was from Peprotech (Changchun, Jilin, China). Antibodies against p65 (sc-372) and p50 (sc-7178) were purchased from Santa Cruz Biotechnology (Changchun, Jilin, China). Goat Anti-Rabbit IgG (111-035-003) was from Jackson ImmunoResearch (Changchun, Jilin, China).

### 4.2. DNA Affinity Precipitation Assays (DAPA)

HEK 293T cells treated with 10 ng/mL of TNFα for 30 min were lysed for 10 min on ice in whole cell lysis buffer (25 mM of 4-(2-hydroxyethyl)-1-piperazineethanesulfonic acid (HEPES) (PH7.7), 300 mM NaCl, 1.5 mM MgCl_2_, 0.2 mM EDTA, 0.5% Triton, 10% glycerol, 2 mM Na_3_VO_4_, 2 mM dl-Dithiothreitol (DTT), 0.5 mM Phenylmethanesulfonyl fluoride (PMSF), and proteinase inhibitors (Roche, Changchun, Jilin, China), and the protein concentration in the supernatants was determined by Coomassie brilliant blue assay. Total 400 µg of proteins were incubated at 4 °C for 30 min with biotin-labeled double-strand oligonucleotide (4 pmol per sample) listed in Table S2, followed by incubation at 4 °C for 1 h with 20 μL of Streptavidin-Agarose. The protein-DNA-biotin-Streptavidin-Agarose complexes were collected by spinning and washed three times with cold Phosphate Buffered Saline (PBS), and then separated by 8% SDS-PAGE. The separated proteins were analyzed by Western blotting using anti-p65 or anti-p50 antibody, respectively.

### 4.3. Plasmid Construction and Mutagenesis

The DNA fragments obtained by annealing of two pairs of single-strand oligonucleotides (FABP6-f/FABP6-r, FABP6-M-f/FABP6-M-r) listed in Table S3 were cloned into MluI/XhoI sites of the pGL3-Promoter vector (Promega, Changchun, Jilin, China), namely P-FABP6 and P-FABP6-Mut, respectively. Human genomic DNA extracted from U937 cells using a Tissue DNA kit (OMEGA BIO-TEK) was used as a template to amplify *CCL2* Promoter and Enhancer fragment by PCR using the primers listed in Table S3. To obtain B-CEP vector, *CCL2* promoter and enhancer fragments were successively cloned into the Nhe1/Xho1 and Kpn1/Nhe1 sites of the pGL3-Basic luciferase reporter vector (Promega) respectively. Plasmid B-CEP-M1M2 with T to C mutations (TGGGAACTTCC to CGGGAACTTCC, and TGGGAATTTCC to CGGGAATTTCC, respectively) was constructed by site-directed mutagenesis with the indicated primers listed in Table S3.

### 4.4. Transfection and Luciferase Reporter System

Methylated plasmids were obtained by incubating the plasmids with CpG methyltransferase M.SssI (M0226, NEB) overnight at 37 °C. DNA methylation was verified by digestion with the methylation-sensitive restriction endonuclease BstUI (R0518, NEB). HEK 293T cells were transfected with the indicated plasmids using the X-tremegene HP (Roche). Twenty-four hours after transfection, the cells were treated with 10 ng/ml of TNFα for 18 hours. NF-κB activation was measured by Luciferase activities using the ONE-Glo™ Luciferase Assay System (Promega).

### 4.5. Bisulfite Sequencing PCR (BSP)

Genomic DNAs from U937, 8226 and 293T cells were extracted using a Tissue DNA kit (OMEGA BIO-TEK, Changchun, Jilin, China), and modified with bisulfite using the EZ DNA methylation-Gold kit (Zymo Research, D5005, Changchun, Jilin, China) according to manufacturers’ protocols. Bisulfite-treated samples were then amplified by PCR using primers listed in Table S4. PCR products were cloned into pMD19 T-Vector (TaKaRa, 6013, Changchun, Jilin, China) and plasmids isolated from randomly picked colonies were sequenced.

### 4.6. Reverse transcription PCR (RT-PCR)

U937 and HEK 293T cells were treated with 0.1 or 10 µM 5-Aza-CdR for 3 days, respectively. Total RNA from cells treated with or without 10 ng/mL of TNFα for the indicated times, was extracted with RNeasy Plus Mini kit (Qiagen, Changchun, Jilin, China). The cDNA was prepared using the GoScript™ Reverse Transcription System (Promega) and used as templates for PCRs amplification of the genes listed in Table S5. PCR fragments were then analyzed by agarose gel electrophoresis.

## Figures and Tables

**Table 1:** Nucleotide at -1 position of κB sites of *VCAM1*, *CCL2* and *RelB* genes (10 kb upstream to and 1 kb downstream transcription start sites) in various vertebrates.

Class	Species	*VCAM1*				*CCL2*				*RelB*										
Mammal	*Homo sapiens*			D	D	D	D	D	D									D	C	
	*Pan troglodytes*			D	D		D	D	D									D	C	
	*Mus musculus*				D		D	D							D	D	D	D	C	
	*Bos taurus*	D	D	D	D		D									D	D	D	C	
	*Canis lupus familiaris*			D	D	D	C	D						D	D	D	D	D	C	
	*Lipotes vexillifer*		D	D	D	D	D	D							D	D	D	D	C	D
	*Eptesicus fuscus*				D		D	C	D							D	D	D	D	
	*Pteropus alecto*				D		D	D							C/D	D	D	D	D	
Aves	*Pseudopodoces humilis*				D					C/D	C/D	D	C	D	D	D	D	D	C	C/D
	*Aquila chrysaetos canadensis*			C/D	C/D													D	C	
Reptilia	*Chrysemys picta*				N														C	
	*Alligator mississippiensis*																		C	
	*Python bivittatus*				D													D	D	
	*Alligator sinensis*				D														N	
Amphhibia	*Xenopus (Silurana) tropicalis*			D	D										D	D	D	D	D	
Pisce	*Notothenia coriiceps*				N														D	
	*Stegastes partitus*				N														C	
	*Poecilia reticulata*			D	D												C	C/D	D	
	*Zebra fish*				D														D	

C, cytosine; D, adenine, thymine or guanine; N, no any κB site; strips, no sequence information available; shadow, in CGIs.

## Supplementary Materials

Supplementary materials can be found at www.mdpi.com/1422-0067/18/3/528/s1.
