# Supplementary material for: The CpG Dinucleotide Adjacent to a κB Site Affects NF-κB Function through Its Methylation
Source: Int J Mol Sci. 2017 Mar 1;18(3):528. doi: 10.3390/ijms18030528 (PMC5372544; doi:10.3390/ijms18030528)
Supplement: Supplementary file 1 [file ijms-18-00528-s001.pdf]

The CpG Dinucleotide Adjacent to A κB Site Affects NF-κB Function through Its Methylation

Tao Wang, Jinge Li, Ke Ding, Li Zhang, Qiuru Che, Xiuming Sun, Yumeng Dai, Wei Sun, Meiying Bao, Xiaochun Wang, Liquan Yang, Zhiwei Li*

The Center for Combinatorial Chemistry and Drug Discovery, School of Pharmaceutical Science, Jilin University, Changchun, China

* Correspondence: zhiweili@jlu.edu.cn; Tel.: +86-431-8561-9260

Table S1: κB sequences and their bearing genes used in this study.

κb site sequence	Human gene name							
GGGAATTTCC	*CCL2*	*NFKBIA*	*CD40*	*NOD2*	*TNFRSF9*	*PLAU*	*CXCL5*	*MUC2*
GGGACTTTCC	*FABP6*	*CD54*	*TNFAIP3*	*LMP2*	*TICAM1*	*TP53*		
GGGATTTCCC	*VCAM1*	*APOC3*	*IL15RA*	*TNIP3*	*BCL3*	*NR4A2*		
GGGGTTTTCC	*CCL20*	*TRPC1*	*CD80*	*RELB*	*LIPG*	*TP53*		
GGGGATTTCC	*SELE*	*TNFAIP3*	*ELF3*	*IRF2*				
GGGGCTTTCC	*CD48*	*VIM*	*PI3*	*NFKB2*				
GGGATTTTCC	*BLR1*	*CD69*	*IL6*					
GGGGAATTCC	*RELB*	*TFPI2*						
GGGAGTTTCC	*CCL5*	*IRF7*						
GGGGCTTCCC	*LTA*	*NFKB1*						
GGGAGATTCC	*FLRG*	*CCL11*						
GGGAATCTCC	*IL2RA*	*GZMB*						
GGGGTTTCCC	*EBI3*	*BLR1*						
GGGGAGCCCC	*CD74*	*IL1A*						
GGGAAACTCC	*CD40*	*BLR1*						
GGGAACTTCC	*CD40*	*CCL2*						
GGGACCCTCC	*GSTP1*							
GGGGAATCCC	*IRF1*							
GGGGATTCCC	*PTGS2*							
GGGACGTTCC	*CREB3*							
GGGATCCTCC	*CD44*							
GGGGAACTCC	*PTX3*							
GGGAATTCCC	*NFKB2*							
GGGGCATCCC	*CCL5*							
GGGGAGTCCC	*EDN1*							
GGGAAATTCC	*IFNB1*							
GGGACATTCC	*LCN2*							
GGGAGGCTCC	*ADAM19*							
GGGAACCTCC	*KCNK5*							
GGGATTCTCC	*BTK*							
GGGACATCCC	*ALOX12*							

Red, -1C κb site; blue, -1C or D κb site; green background, κb site in CGI.

Table S2. Oligonucleotides used for DAPA

Oligonucleotide	Sequence (5'→3')
FABP6 κb site -1A	Biotin-CCTCTTCAAAGGGACTTTCCTACAAGGGACTTTCCTTCCCGTCTA<br>TAGACGGGAAGGAAAGTCCCTTGTAGGAAAGTCCCTTTGAAGAGG
FABP6 κb site -1C	Biotin-CCTCTTCAACGGGACTTTCCTACACGGGACTTTCCTTCCCGTCTA<br>TAGACGGGAAGGAAAGTCCCGTGTAGGAAAGTCCCGTTGAAGAGG
FABP6 κb site -1 5mC	Biotin-CCTCTTCAA/5mC/GGGACTTTCCTACA/5mC/GGGACTTTCCTTCCCGTCTA<br>TAGACGGGAAGGAAAGTCC/5mC/GTGTAGGAAAGTCC/5mC/GTTGAAGAGG
CCL2 κb site -1A	Biotin-CCTCTTCAAAGGGAACTTCCTACAAGGGAATTTCCTTCCCGTCTA<br>TAGACGGGAAGGAAATTCCCTTGTAGGAAGTTCCCTTTGAAGAGG
CCL2 κb site -1C	Biotin-CCTCTTCAACGGGAACTTCCTACACGGGAATTTCCTTCCCGTCTA<br>TAGACGGGAAGGAAATTCCCGTGTAGGAAGTTCCCGTTGAAGAGG
CCL2 κb site -1 5mC	Biotin-CCTCTTCAA/5mC/GGGAACTTCCTACA/5mC/GGGAATTTCCTTCCCGTCTA<br>TAGACGGGAAGGAAATTCC/5mC/GTGTAGGAAGTTCC/5mC/GTTGAAGAGG
PTX3 κb site -1C	Biotin-CCTCTTCAAAGGGGAACTCCCGTACAAGGGGAACTCCCGTTCCCGTCTA<br>TAGACGGGAACGGGAGTTCCCCTTGTACGGGAGTTCCCCTTTGAAGAGG
PTX3 κb site -1 5mC	Biotin-CCTCTTCAAAGGGGAACTCC/5mC/GTACAAGGGGAACTCC/5mC/GTTCCCGTCTA<br>TAGACGGGAA/5mC/GGGAGTTCCCCTTGTA/5mC/GGGAGTTCCCCTTTGAAGAGG
FLRG κb site -1C	Biotin-CCTCTTCAAAGGGAGATTCCCGTACAAGGGAGATTCCCGTTCCCGTCTA<br>TAGACGGGAACGGGAATCTCCCTTGTACGGGAATCTCCCTTTGAAGAGG
FLRG κb site -1 5mC	Biotin-CCTCTTCAAAGGGAGATTCC/5mC/GTACAAGGGAGATTCC/5mC/GTTCCCGTCTA<br>TAGACGGGAA/5mC/GGGAATCTCCCTTGTA/5mC/GGGAATCTCCCTTTGAAGAGG
NC	Biotin-CCTCTTCAAATTGAACTTACTACAATTGAACTTACTTCCCGTCTA<br>TAGACGGGAAGTAAGTTCAATTGTAGTAAGTTCAATTTGAAGAGG

Table S3. Primers used for Plasmids construction and site-directed mutagenesis

Primer	Sequence (5'→3')
FABP6- f	CGCGTCCTCTTCAAAGGGACTTTCCTACAAGGGACTTTCCTTCCCGTCTAC
FABP6- r	TCGAGTAGACGGGAAGGAAAGTCCCTTGTAGGAAAGTCCCTTTGAAGAGGA
FABP6-M-f	CGCGTCCTCTTCAACGGGACTTTCCTACACGGGACTTTCCTTCCCGTCTAC
FABP6-M-r	TCGAGTAGACGGGAAGGAAAGTCCCGTGTAGGAAAGTCCCGTTGAAGAGGA
CCL2-E-f	ACGTGGTACCGTGTGTCCCCAAGCGAG
CCL2-E-r	GATCGCTAGCTGCATCCTTTACCATGAACT
CCL2-P-f	GATCGCTAGCAATTCAGTTCAATGTTTACA
CCL2-P-r	AGTCCTCGAGGCTGGAGGCGAGAGTGCGAG
CCL2-Mut1-f	GCATTCTCTTCTACGGGATCCGGGAACTTCCAAAGCTGC
CCL2-Mut1-r	GCAGCTTTGGAAGTTCCCGGATCCCGTAGAAGAGAATGC
CCL2-Mut2-f	CAAAGCTGCCTCCTCAGAGCGGGAATTTCCACTCACTTC
CCL2-Mut2-r	GAAGTGAGTGGAAATTCCCGCTCTGAGGAGGCAGCTTTG

Table S4. Primers used for BSP

Primers used for BSP	
Primer	Sequence (5'→3')
GSTP1-M-f	TTTGTTGTTTGTTTATTTTTTAGGTTT
GSTP1-M-r	ATACTAAAAACTCTAAACCCCATCC
RelB-M-f	GTTTAAGTTTTATTGGGAGATTAAAG
RelB-M-r	ACCCAAAAACTAACCCAAACC
IRF7-M-f	GGGTTTTTAGTATTTGGGTGTTAGAG
IRF7-M-r	AAACTATAATAAAATAACTCCATCTC
TNFIP3-M-f	GYGGGGTAGGGAAAGG
TNFIP3-M-r	AACTCCAAACTCRCTTAACC
TFPI2-M-f	TTTTATGTTTTTAAGAGGTGGATTT
TFPI2-M-r	AAACCTAAAAAATAACTAATTCATACAC
PTX3-M-f	GATTTTTTTTTAATTAATTTGATTGTAG
PTX3-M-r	AAATCCTTAATAAATACTAAAAAAAACC
FLRG-M-f	TTTTGTGTTTGTTTTATTTTTTAAGT
FLRG-M-r	CCTAACCTTAACCTCTAACATTTCC
CCL2-M-f	TTTTTATAGTTTTTTTGGGGGTTTT
CCL2-M-r	ATCTACCTCCCACTTCTACTCTATCAA

Table S5. Primers used for RT-PCR

Primer	Sequence (5'→3')
RelB-T-f	GCCTCGTGGGGAAAGACT
RelB-T-r	TGTCGCAGAGCAAGTAGAGC
TNFAIP3-T-f	ACCCCATTGTTCTCGGCTAT
TNFAIP3-T-r	CCCTGCTCGCTGTTTTCC
CCL2-T-f	TAGCAGCCACCTTCATTCC
CCL2-T-r	GCTTGGGGTCAGCACAGAT
IκBα-T-f	AGTACGAGCAGATGGTCAAG
IκBα-T-r	TCATGGATGATGGCCAAGTG
TFPI2-T-f	CCCTACTTCTCCGTTACTACT
TFPI2-T-r	TCTGGAAACCTGTTCTCAAT
IRF7-T-f	CAGATCCAGTCCCAACCAA
IRF7-T-r	GCAGCAGTTCCTCCGTGT
GAPDH-T-f	AGCCACATCGCTCAGACACC
GAPDH-T-r	ACGTACTCAGCGCCAGCATC

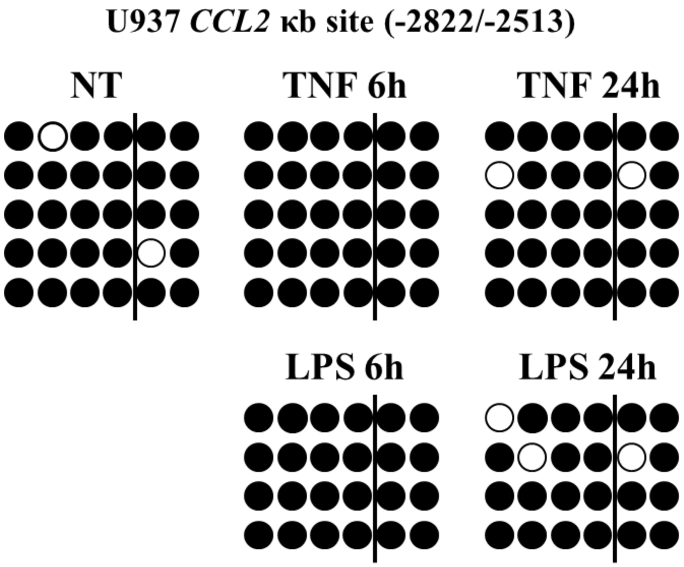

Figure S1. Methylation status around the κB sites of *CCL2* was not changed upon NF-κB activation by TNFα or LPS in U937 cells. Genomic DNAs were isolated from U937 cells treated with TNFα (10 ng/ml) or LPS (1μg/ml) for the indicated time and methylation status was determined by BSP. The κB site was shown as a vertical line.

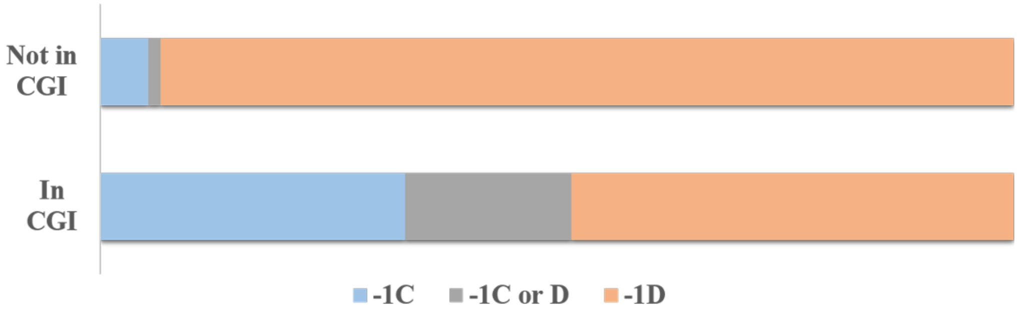

Figure S2. The ratio between -1C κB sites and total κB sites outside CGIs is substantially lower than that inside CGIs in *VCAM*, *CCL2* and *RelB* of multiple vertebrate.
